# Supplementary material for: Ectopic engraftment of nociceptive neurons derived from hPSCs for pain relief and joint homeostasis
Source: bioRxiv. 2025 Dec 19:2025.12.16.694733. Preprint. [Version 1] doi: 10.64898/2025.12.16.694733 (PMC12746131; doi:10.64898/2025.12.16.694733)
Supplement: Supplement 1 [file NIHPP2025.12.16.694733v1-supplement-1.pdf]

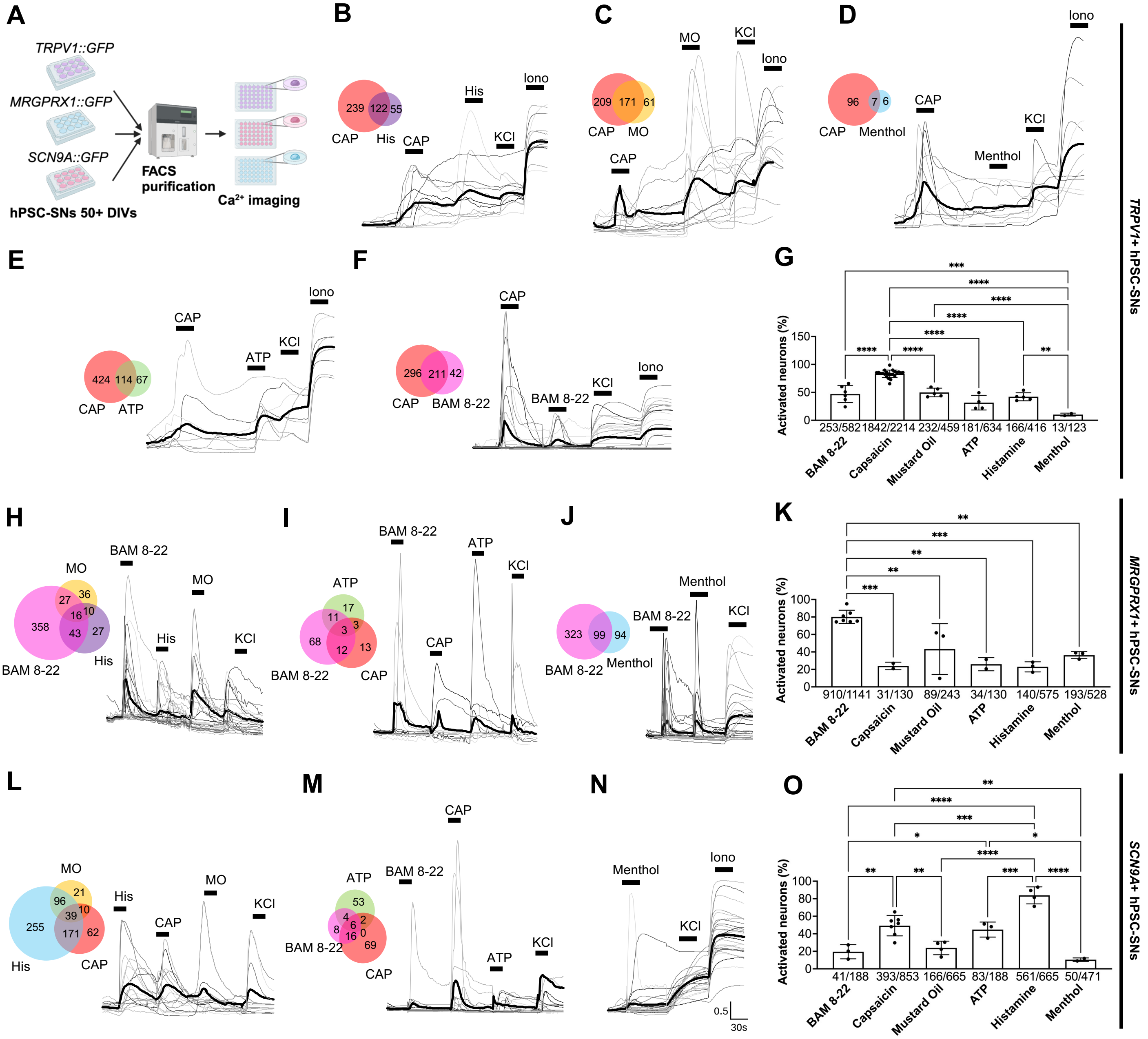

Figure S1. *SCN9A+, TRPV1+* nociceptors and *MRGPRX1+* pruriceptors are polymodal and respond to canonical noxious or pruritogenic stimuli, related to Figure 1.
(A) Workflow of FACS purification of *MRGPRX1+, SCN9A+ and TRPV1+* hPSC-SNs followed by Ca^{2+} imaging.

(B-G) Representative Ca^{2+} transients of FACS-purified *TRPV1+* hPSC-SNs in response to capsaicin (10μM; **B-F**), histamine (50μM; **B**), mustard oil (100μM; **C**), menthol (100μM; **D**), ATP (50μM; **E**), and BAM8-22 (10μM; **F**); bold traces show the mean response. Venn diagrams show the overlap in stimulus responsiveness. Quantification of proportion of *TRPV1+* neurons activated by each stimuli **(G)** (n ≥ 3 independent differentiations; one-way ANOVA with Tukey's multiple comparisons test; ** $P < 0.01$, *** $P < 0.001$, **** $P < 0.0001$; mean ± s.e.m. with individual values overlaid).

(H-K) Representative Ca^{2+} transients of FACS-purified *MRGPRX1+* hPSC-SNs in response to capsaicin (10μM; **I**), histamine (50μM; **H**), mustard oil (100μM; **H**), menthol (100μM; **J**), ATP (50μM; **I**), and BAM8-22 (10μM; **H-J**); bold traces show the mean response. Venn diagrams show the overlap in stimulus responsiveness. Quantification of proportion of *MRGPRX1+* neurons activated by each stimuli **(K)** (n ≥ 3 independent differentiations; one-way ANOVA with Tukey's multiple comparisons test; ** $P < 0.01$, *** $P < 0.001$; mean ± s.e.m. with individual values overlaid).

(L-O) Representative Ca^{2+} transients of FACS-purified *SCN9A+* hPSC-SNs in response to capsaicin (10μM; **L,M**), histamine (50μM; **L**), mustard oil (100μM; **L**), menthol (100μM; **N**), ATP (50μM; **M**), and BAM8-22 (10μM; **M**); bold traces show the mean response. Venn diagrams show the overlap in stimulus responsiveness. Quantification of proportion of *SCN9A+* neurons activated by each stimuli **(O)** (n ≥ 3 independent differentiations; one-way ANOVA with Tukey's multiple comparisons test; * $P < 0.05$, ** $P < 0.01$, *** $P < 0.001$, **** $P < 0.0001$; mean ± s.e.m. with individual values overlaid).

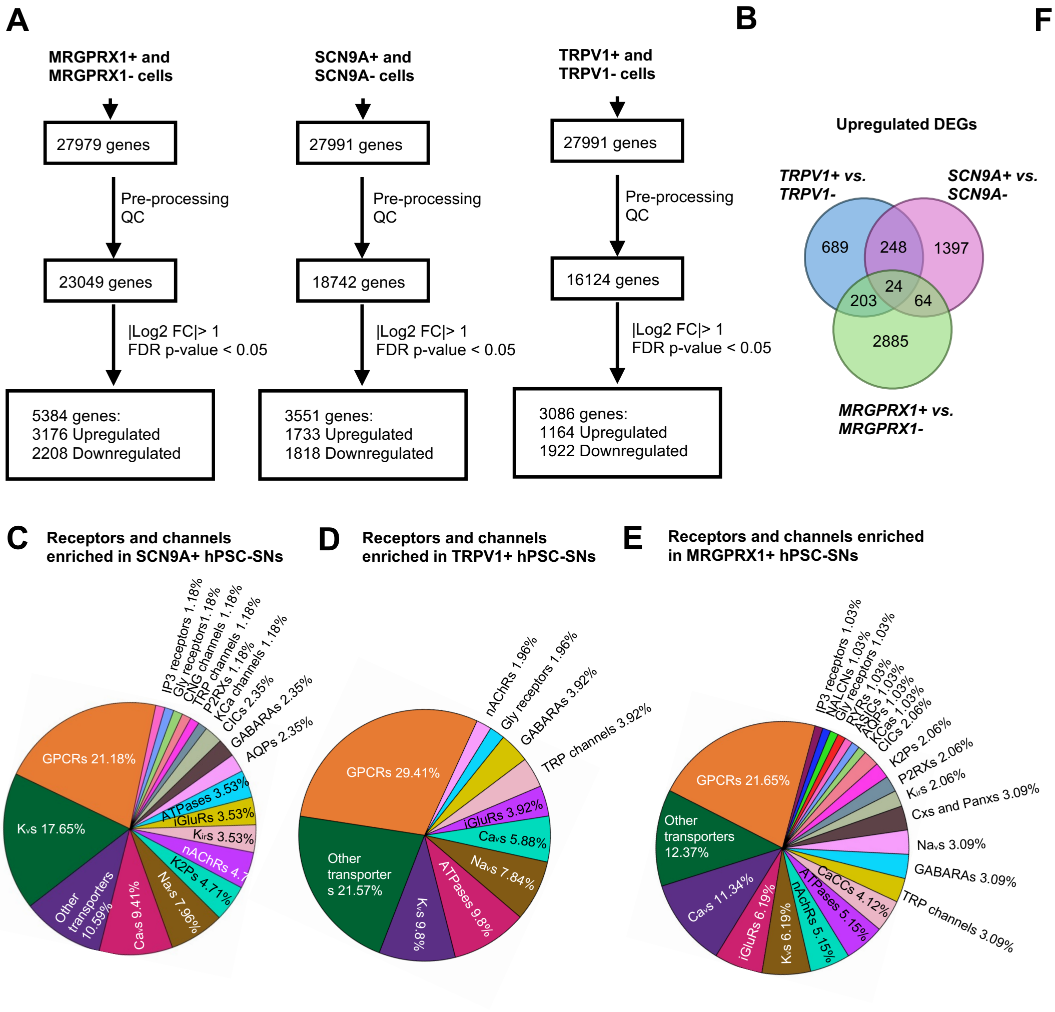

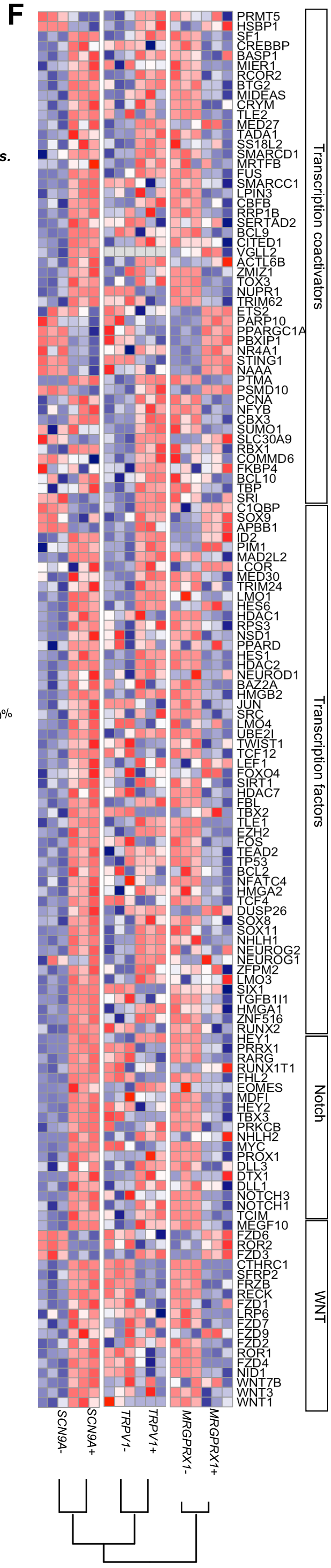

Figure S2. Transcriptomic characterization of *SCN9A+, TRPV1+* nociceptors and *MRGPRX1+* pruriceptors, related to Figure 2.

(A) Workflow of purifying GFP+ and GFP- cells from hPSC-SN cultures of *MRGPRX1::GFP, SCN9A::GFP*, and *TRPV1::GFP* reporter lines (n = 5 independent differentiations; n = 3 technical replicates; $\log_2$FC cutoff = 1; P cutoff = 0.05).

(B) Venn diagram shows significantly enriched genes in *MRGPRX1+, SCN9A+ and TRPV1+* hPSC-SNs compared to their negative counterparts, highlighting shared and lineage-specific genes.

(C-E) Pie charts categorizing enriched receptors and ion channels enriched in *SCN9A+* **(C)**, *TRPV1+* **(D)** and *MRGPRX1+* **(E)** hPSC-SNs.

(F) GSEA on differentially expressed transcription factors and key pathway proteins between GFP+ and GFP- populations in each reporter line using MSigDB gene sets; hierarchical clustering of pairwise lineage comparisons is shown as a dendrogram.

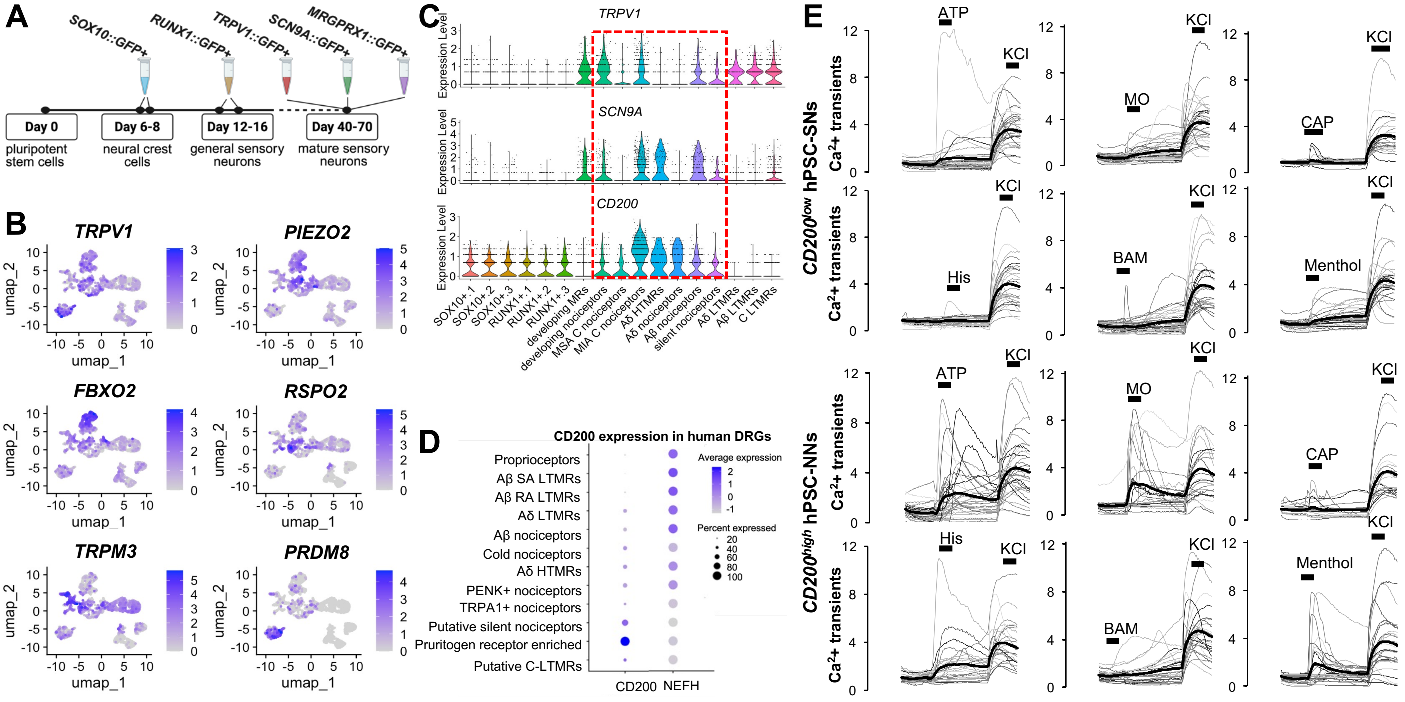

Figure S3. Transcriptomic and functional analyses reveal specific enrichment of *CD200* in nociceptors, related to Figure 3.

(A) Workflow for constructing a transcriptomic continuum of nociceptor development from FACS-purified *SOX10::GFP*+ neural crest cells, *RUNX1::GFP*+ sensory progenitor cells, *MRGPRX1::GFP*+, *SCN9A::GFP*+, and *TRPV1::GFP*+ hPSC-SNs by scRNA-seq (≈ 1,000 cells per lineage; n = 3 independent differentiations).

(B) Feature plots on the UMAP embedding showing expression of additional marker genes used to assign each cluster identity.

(C) Violin plots showing the co-enrichment of *TRPV1*, *SCN9A* and *CD200* in nociceptor clusters.

(D) Dot plot showing *CD200* enrichment in small-fiber nociceptors form a published human DRG neuron single-cell dataset[7].

(E) Representative Ca^{2+} transients demonstrating heightened responses of *CD200*^{high} hPSC-NNs versus *CD200*^{low} hPSC-SNs to ATP (50μM), mustard oil (100μM), capsaicin (10μM), and histamine (50μM); bold traces show the mean response; n ≥ 3 independent differentiations. The response rate to BAM8-22 (10 μM) and menthol (100 μM) did not show significant differences.

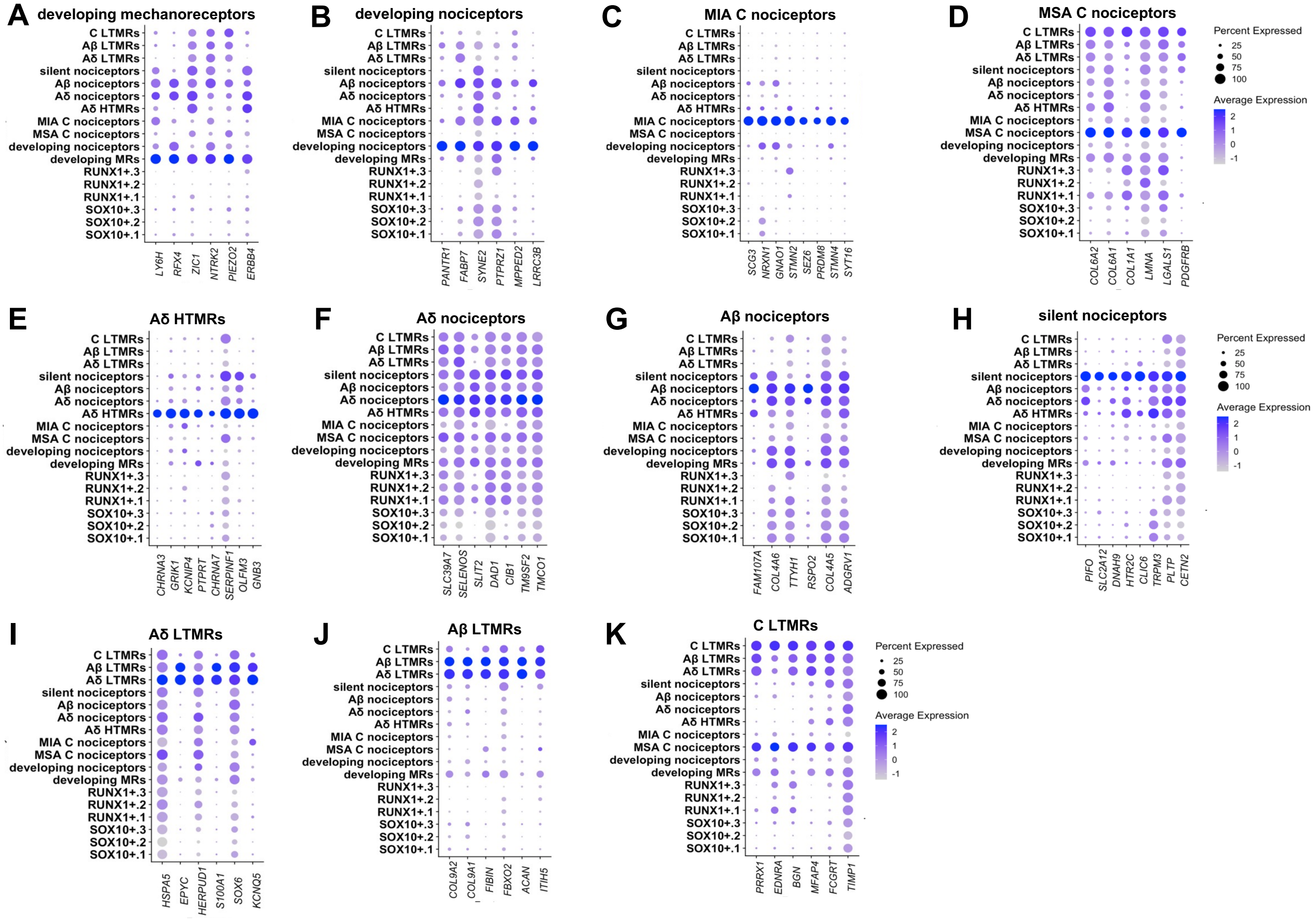

Figure S4. Marker gene expression across hPSC-SNs clusters, related to Figure 2.

(A-K) Dot plots of the top marker genes for each identified neuronal subpopulation, showing their expression across all clusters; dot size reflects the fraction of cells within each cluster expressing the gene, and dot color indicates the mean scaled expression level.

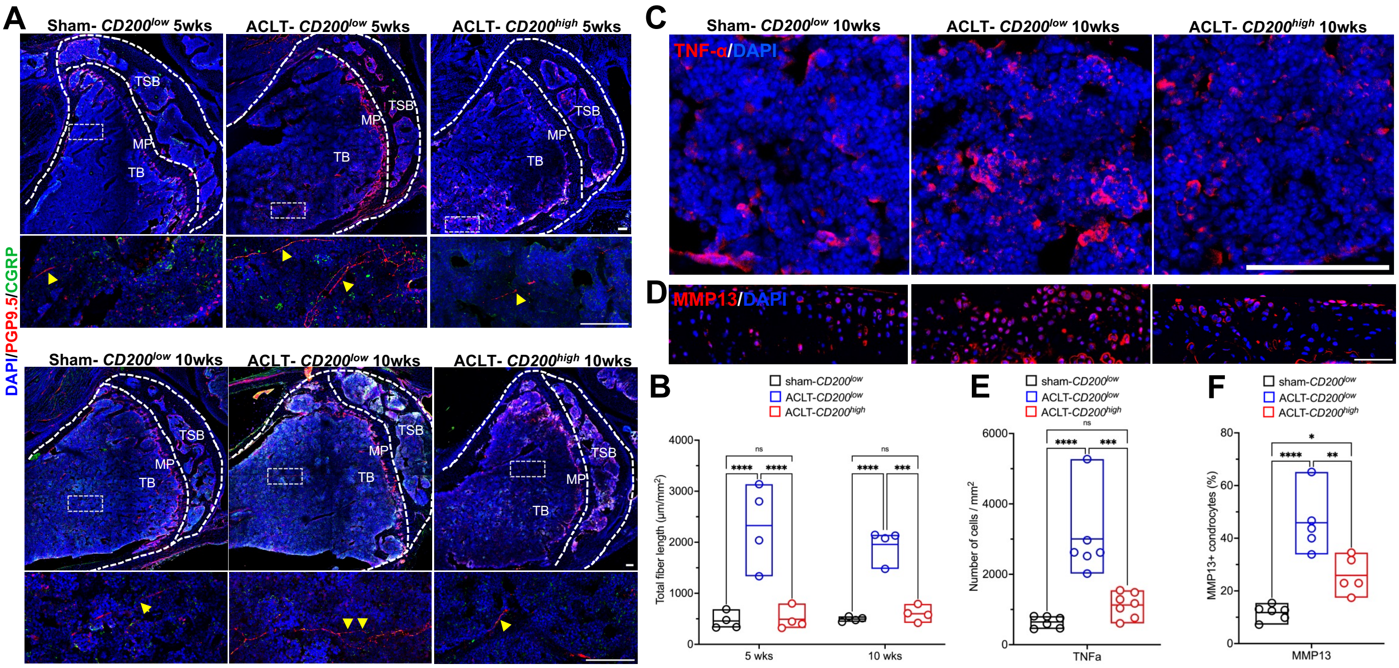

Figure S5. *CD200^high* hPSC-NNs modulate the neuro-immune axis in ACLT- *CD200^high* versus ACLT- *CD200^low* animals, related to Figure 4.

(A) PGP9.5 (red) and CGRP (green) immunostaining in knee sections from sham-*CD200^low*, ACLT-*CD200^low* and ACLT-*CD200^high* animals 5 (top) and 10 (bottom) weeks post-injection; scale bars, 100µm. TSP, tibial subchondral plate; MP, metaphysis; TB, tibial bone.

(B) Quantification of total PGP9.5+ nerve fiber length 5 and 10 weeks post-injection (n ≥ 4 animals; two-way ANOVA with Tukey's test; *** $P < 0.001$, **** $P < 0.0001$; mean ± range with individual values overlaid).

(C,E) TNF-α (red) immunostaining in sham-*CD200^low*, ACLT-*CD200^low* and ACLT-*CD200^high* joints 10 weeks post-injection; scale bar, 100µm. Quantification of TNF-α expression **(E)** (n ≥ 6 animals; one-way ANOVA with Tukey's test; *** $P < 0.001$, **** $P < 0.0001$; mean ± range with individual values overlaid).

(D,F) MMP13 (red) immunostaining in sham-*CD200^low*, ACLT-*CD200^low* and ACLT-*CD200^high* joints 10 weeks post-injection; scale bar, 100µm. Quantification of MMP13 expression **(F)** (n ≥ 5 animals; one-way ANOVA with Tukey's test; * $P < 0.05$, ** $P < 0.01$, **** $P < 0.0001$; mean ± range with individual values overlaid).

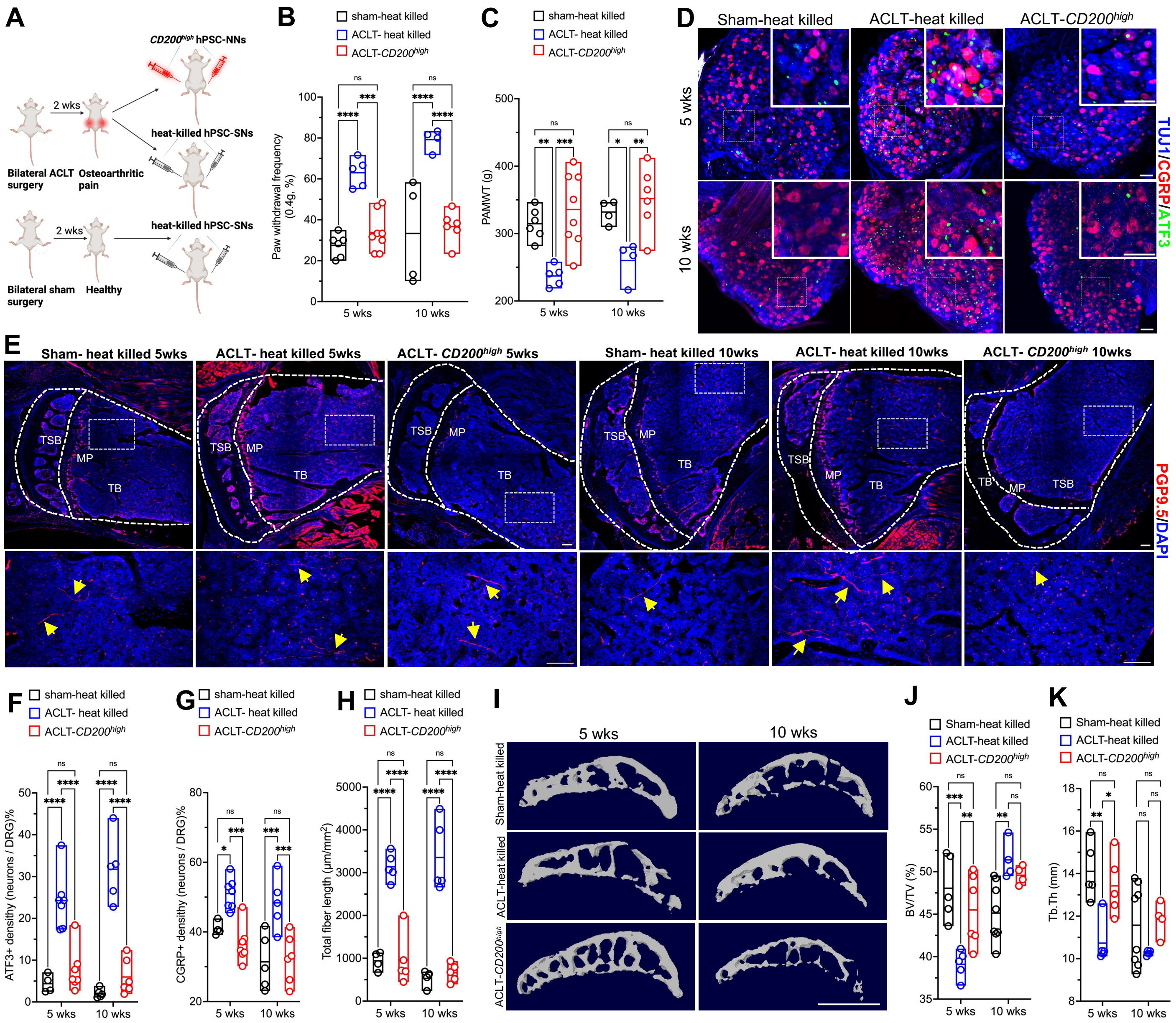

Figure S6. *CD200^{high}* hPSC-NNs reduce pain and improve subchondral microarchitecture in ACLT- *CD200^{high}* versus ACLT-heat killed animals, related to Figure 4.

(A) Experimental timeline of bilateral intra-articular and tibia tuberosity injections of *CD200^{high}* hPSC-NNs or heat-killed hPSC-SNs in NIH-III mice two weeks after sham or ACLT surgery.

(B-C) Mechanical hypersensitivity assessed by Von Frey **(B)** and PAMWT **(C)** in sham-heat killed, ACLT-heat killed and ACLT-*CD200^{high}* animals 5 and 10 weeks post-injection (n ≥ 4; two-way ANOVA with Tukey's multiple comparisons test; * P < 0.05, ** P < 0.01, *** P < 0.001, **** P < 0.0001; mean ± range with individual values overlaid).

(D,F,G) L3–L4 whole DRGs immunostained for ATF3 (green) and CGRP (red) at 5 and 10 weeks; scale bars, 100μm. Quantification of ATF3+ **(F)** and CGRP+ **(G)** neurons in whole DRGs (n ≥ 5; two-way ANOVA with Tukey's test; *** P < 0.001, **** P < 0.0001; mean ± range with individual values overlaid).

(E) PGP9.5 immunostaining (red, arrows) in knee sections from sham-heat killed, ACLT-heat killed and ACLT-*CD200^{high}* animals 5 (left) and 10 (right) weeks post-injection; scale bars, 100μm. TSP, tibial subchondral plate; MP, metaphysis; TB, tibial bone.

(H) Quantification of total PGP9.5+ nerve fiber length 5 and 10 weeks post-injection (n ≥ 4 animals; two-way ANOVA with Tukey's test; **** P < 0.0001; mean ± range with individual values overlaid).

(I-K) Representative microCT of tibia subchondral bone **(I)** and quantification of trabecular bone volume fraction (BV/TV; **J**) and trabecular thickness (Tb.Th; **K**) (n ≥ 5; two-way ANOVA with Tukey's test; * P < 0.05, ** P < 0.01, *** P < 0.001; mean ± range with individual values overlaid).
